# Al-Doped SrMoO_3_ Perovskites as Promising Anode Materials in Solid Oxide Fuel Cells

**DOI:** 10.3390/ma15113819

**Published:** 2022-05-27

**Authors:** Vanessa Cascos, María Teresa Fernández-Díaz, José Antonio Alonso

**Affiliations:** 1 Instituto de Ciencia de Materiales de Madrid, Consejo Superior de Investigaciones Científicas, Cantoblanco, E-28049 Madrid, Spain; jaalonso@icmm.csic.es; 2 Departamento de Química Inorgánica, Universidad Complutense de Madrid, E-28040 Madrid, Spain; 3 Institut Laue Langevin, BP 156X, F-38042 Grenoble, France; ferndiaz@ill.eu

**Keywords:** anode, scheelite, IT-SOFC, SrMoO_3_, perovskite, neutron diffraction

## Abstract

Two perovskite materials with SrMo_1−x_Al_x_O_3−_*_δ_* (x = 0.1, 0.2) compositions have been synthesized by reduction from the corresponding scheelite phases, with SrMo_1−x_Al_x_O_4−_*_δ_* stoichiometry; the pertinent characterization shows that the defective perovskites can be used as anode materials in solid oxide fuel cells, providing maximum output power densities of 633 mW/cm^2^ for x = 0.2. To correlate structure and properties, a neutron powder diffraction investigation was carried out for both perovskite and scheelite phases. Both perovskites are cubic, defined in the *Pm*-3*m* space group, displaying a random distribution of Mo and Al cations over the 1*a* sites of the structure. The introduction of Al at Mo positions produced conspicuous amounts of oxygen vacancies in the perovskite, detected by neutrons. This is essential to induce ionic diffusion, providing a mixed ionic and electronic conduction (MIEC), since in MIEC electrodes, charge carriers are combined in one single phase and the ionic conductivity can be one order of magnitude higher than in a conventional material. The thermal expansion coefficients of the reduced and oxidized samples demonstrated that these materials perfectly match with the La_0.8_Sr_0.2_Ga_0.83_Mg_0.17_O_3−*δ*_ electrolyte, La_0.4_Ce_0.6_O_2−*δ*_ buffer layer and other components of the cell. Scanning electron microscopy after the test in a real solid oxide fuel cell showed a very dense electrolyte and porous electrodes, essential requirements for this type of fuel. SrMo_1−x_Al_x_O_3−*δ*_ perovskites are, thus, a good replacement of conventional biphasic cermet anodes in solid oxide fuel cells.

## 1. Introduction

A solid oxide fuel cell (SOFC) is an electrochemical device totally constituted by solid materials, able to convert the chemical energy of a fuel (typically hydrogen) into electrical power, owing to the ion–conducting properties of the ceramic oxide material of its electrolyte. After reduction of O_2_ from the air to O^2−^ ions in the cathode material, those diffuse through the electrolyte reaching the anode, where they react with the fuel while delivering electricity to an external circuit [1,2]. Normally, SOFC devices work at high temperatures (900–1000 °C) required to stimulate the ionic conduction of oxide ions through the electrodes and electrolyte, which involves issues related to the chemical and mechanical stability of the ensemble [3]. In order to solve these problems and make them more economically viable devices, it is desirable to reduce the working temperature to the 750–850 °C range without deteriorating the operational functions of the cell [4,5]. The performance of these devices is highly affected by the fuel-oxidation reaction, which occurs at the anode electrode. This is conventionally constituted by byphasic cermets, composed of Ni particles mixed with the electrolyte, where the wanted reduction in temperature can drastically diminish the activity. Therefore, the search of anode materials with a better catalytic activity is a prime target. Single phase mixed ionic-electronic conductors (MIECs) are materials where the triple phase boundary (TPB) is enhanced. In these materials, the sites where oxygen ions and the fuel coincide are maximized, increasing the anodic efficiency, and decreasing the anode polarisation resistance [2]. Many of these MIEC materials present a perovskite structure with ABO_3_ formulae, where the doping in both A and B positions with lower oxidation state elements would introduce oxygen vacancies and enhance the ionic transport properties [6,7]. In particular, SrMoO_3_ perovskite presents, itself, a high electronic conductivity of ∼10^5^ Scm^−1^ at 300 K [8,9], in addition to a great redox stability [10]. The partial chemical replacement in this material of Mo atoms with an aliovalent M element (in SrMo_1−x_M_x_O_3−*δ*_) will introduce some oxygen vacancies in the perovskite structure, thus obtaining a suitable MIEC oxide as anode for SOFC. The extraordinary activity of Mo in the hydrogen oxidation reaction (HOR) is well known [11,12,13], hence predicting a good performance of these Mo perovskites.

In previous works in our group, SrMoO_3_ was doped at Mo position with different compositions of different elements such us Fe, Cr, Mg, Ga and Co [14,15,16,17,18], all were able to adopt the B positions of the perovskite structure. These oxides worked properly as anode materials, successfully replacing the Ni-cermets, providing excellent output powers in single SOFCs from 700 to 910 mW cm^−2^ under pure H_2_ at 850 °C. In the present work, Al^3+^ has been chosen to replace Mo at 10% and 20%, due to its great abundance and its capability to adopt an octahedral coordination in a perovskite structure. Since Al^3+^ valence state is not variable and it is lower than the expected tetravalent state for Mo, its incorporation into the lattice is expected to be accompanied with the creation of oxygen vacancies, due to electroneutrality factors.

SrMo_0.9_Al_0.1_O_3−*δ*_ and SrMo_0.8_Al_0.2_O_3−*δ*_ perovskites have been synthesized under reducing conditions and characterized by pertinent techniques, focusing the performance of a single test cell for x = 0.2. A soft-chemistry procedure (citrate method) was carried out to obtain porous oxides, and different properties such us dilatometric analysis, chemical compatibility, redox reversibility, electrical conductivity, and porosity were measured. Finally, a complete structural study including X-ray diffraction (XRD) and neutron powder diffraction (NPD) data were necessary to correlate the structure of the materials and its properties with the performance of the cell.

## 2. Experimental

### 2.1. Synthesis

Scheelites of SrMo_0.9_Al_0.1_O_4−*δ*_ and SrMo_0.8_Al_0.2_O_4−*δ*_ (SMAO) compositions were prepared using a soft chemistry synthetic route: the citrate-nitrate method. Stoichiometric amounts of Sr(NO_3_)_2_, (NH_4_)_6_Mo_7_O_24_·H_2_O and Al(NO_3_)_2_·9H_2_O were weighted and dissolved in a 10% solution of citric acid under constant heating at 60 °C and stirring. After evaporation, the resulting resins were dried at 120 °C for 3 h and calcined at 600 °C for 12 h in air. The final samples were obtained after the reduction of the scheelite powders by placing the samples into a tubular furnace and heating them at 1050 °C for 15 h under H_2_/N_2_ (5%/95%) flow. The use of this reduced atmosphere allows the reduction of Mo^6+^ to Mo^4+^, yielding the wanted SrMo_0.9_Al_0.1_O_3−*δ*_ and SrMo_0.8_Al_0.2_O_3−*δ*_ perovskites.

### 2.2. Structural Characterization

The characterization of these oxides was first achieved using powder XRD at room temperature, to corroborate pure phases and assess the introduction of Al in the material. A Bruker D8 Advanced diffractometer (40 kV, 30 mA, Karlsruhe, Germany), controlled by a DIFFRACT^PLUS^ software (Germany), in a Bragg-Brentano configuration with CuK_α_ (λ = 1.5418 Å) radiation was used for this purpose. XRD data were collected in the 2θ range of 11–64° with a 0.02° step. In addition, an NPD study at 25 °C for SrMo_1−x_Al_x_O_3−_*_δ_* (x = 0.1, 0.2) samples was carried out at the D2B diffractometer of the ILL (Grenoble), with wavelength λ = 1.594 Å in order to determine the oxygen content in the perovskites and calculate the level of Al doping in these materials. An amount of 2 g of the samples was packed in a cylindrical vanadium holder (dia. 8 mm), and the counting time was 2 h in the high-intensity mode. Finally, the thermal evolution of the x = 0.2 sample was investigated at increasing temperatures (300, 600, 850 °C) in a furnace with V resistors, working under 10^−4^ mbar vacuum, coupled to the D2B diffractometer. The coherent scattering lengths for the elements contained in the sample are: Sr (7.020 *fm*), Mo (6.715 *fm*), Al (3.449 *fm*), and O (5.803 *fm*) [19]. The Fullprof software was used to refine the structure [20] with the Rietveld method [21]. The zero-point error, scale factor, background coefficients, pseudo-Voigt shape parameters, occupancy of the elements, atomic coordinates, and anisotropic displacements for the O atoms were refined.

### 2.3. Thermal Expansion Coefficients

Dilatometry experiments were performed on sintered pellets of the perovskite and scheelite phases. A Linseis L75HX1000 dilatometer (Selb, Germany) was utilized, between 200 and 850 °C in H_2_/N_2_ (reduced phases) or air (oxidized phases) flow with a heating rate of 10 °C min^−1^. Perovskite pellets of around 7 mm of diameter and 1.5 mm of thickness were prepared by uniaxial pressing of the powders and subsequently annealed at 1050 °C for 12 h in H_2_/N_2_ (5%/95%) flow in order to prevent the oxidation of the material. On the other hand, pellets of the scheelite powders were sintered at 1050 °C for 12 h in air.

### 2.4. Thermal Analysis

Thermogravimetric (TG) curves were collected in O_2_ flow using a Mettler TA-3000 system (Barcelona, Spain) equipped with a TC10 processor unit, starting from the reduced perovskite phases. The curves were obtained in a TG50 unit heating from 30 to 900 °C with a heating rate of 10 °C min^−1^ using approximately 40 mg of sample.

### 2.5. DC-Conductivity

The electrical conductivity was measured in sintered rectangular bars (∼2 mm × 3mm × 9 mm) in the temperature range between 20 and 850 °C, using the *dc* four-probe technique under *dc* currents between 0.01 and 0.5 A. The rectangular bars were prepared using a Retsch PP25 Hydraulic Press (Haan, Germany), pressing the powders and heating them at 1050 °C for 12 h under a H_2_/N_2_ (5%/95%) atmosphere. Finally, four Pt wires were added to the pellets in a four-point configuration with Pt paste and calcined at 850 °C for 1 h under H_2_/N_2_ flow. A Potentiostat-Galvanostat AUTOLAB PGSTAT 302 from ECO CHEMIE (Utrecht, The Netherlands) was used to measure the electrical conductivity of the samples collected every 50 °C under H_2_/N_2_ flow.

### 2.6. Single-Cell Performance

SrMo_0.8_Al_0.2_O_3−*δ*_ (SMAO) was tested as an anode material in single cells in an electrolyte-supported configuration, using La_0.8_Sr_0.2_Ga_0.83_Mg_0.17_O_3−*δ*_ (LSGM) as an electrolyte (15 mm in diameter) and SrCo_0.8_Fe_0.2_O_3−*δ*_ (SCFO) as a cathode. The LSGM material was obtained from the starting materials (La_2_O_3_, SrCO_3_, Ga_2_O_3_ and MgO), following a ceramic method, with sequential heating treatments in air at 1000 °C and 1200 °C for 20 h in air, with intermediate grinding. The final LSGM pellets were obtained by pressing LSGM powders under 200 MPa and then sintering them at 1450 °C in air for 20 h at a ramping rate of 3 °C min^−1^. After grinding the pellets with rotating SiC wheels, the resulting LSGM pellets had a thickness of around 0.3 mm.

Single cells with configuration of SMAO|LDC|LSGM|SCFO were evaluated, where LDC (La_0.4_Ce_0.6_O_2−*δ*_) played a buffer layer role and was deposited between the electrolyte and anode in order to avoid phase interdiffusion in the heating period. A slurry consisting of SMAO, LDC, and SCFO powders and a Heraeus binder V006 was sequentially applied to both surfaces of the electrolyte. LDC ink was screen-printed onto one side of the LSGM disk followed by a thermal treatment at 1300 °C in air for 1 h, and SMAO was subsequently screen printed onto the LDC layer and fired at 1100 °C in air for 1 h. Finally, SCFO was screen printed onto the other side of the disk and fired at 1100 °C in air for 1 h. The resulting electrodes had a thickness of 5 μm and an effective electrode area of 0.25 cm^2^. Platinum gauze was deposited and pasted with Pt paste on the anode and cathode side as current collector. The single cell (with the anode side exposed to H_2_, flowing at a rate of 20 mL min^−1^ during the measurements) was deposited on top of an alumina tube and sealed with a glass-ceramic sealant in order to avoid the admission of air in the system. The cathode side was open to the air. The cells were tested in a vertical tubular furnace at 800 and 850 °C. Electrochemical characterizations of the cells were performed under ambient pressure using an AUTOLAB 302N Potentiostat/Galvanostat by changing the voltage of the cell from 1.2 to 0.1 V, with steps of 0.010 V, holding 10 s at each step. Current density was calculated by the recorded current flux through the effective area of the cell (0.25 cm^2^). Each VI (voltage-intensity) scan corresponds to one cycle; the activation of the cell was followed in subsequent cycles until the full power of the single cell was reached.

### 2.7. Scanning Electron Microscopy

Scanning electron microscopy (SEM) images of a post-mortem cross section of the cell were collected with a table-top Hitachi TM-1000 microscope (Tokyo, Japan). Field-effect scanning electron microscopy (FE-SEM) images of the anode material were obtained in a FEI Nova microscope, with an acceleration potential of 5 kV, coupled to an energy-dispersive X-ray spectrometer (EDX), working with an acceleration voltage of 18 kV and 60 s of acquisition time.

## 3. Result and Discussion

### 3.1. Crystallographic Characterization

**Figure 1:**
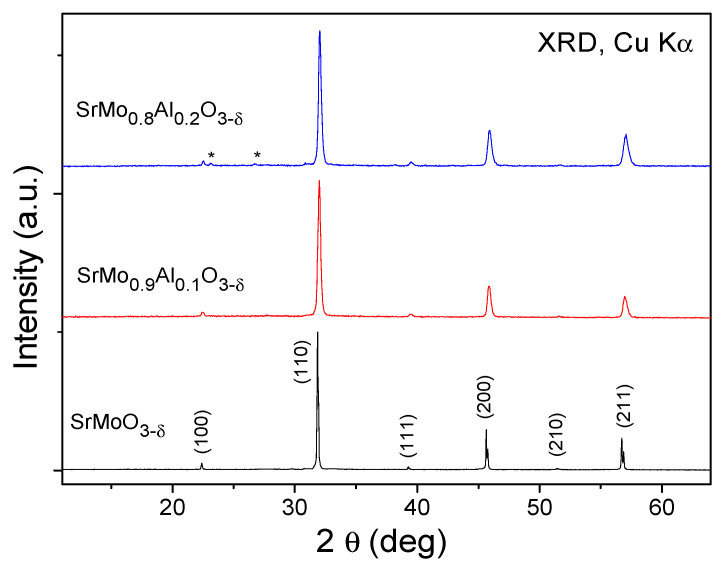
The XRD patterns with CuK_α_ radiation for SrMo_1__−__x_Al_x_O_3__−*δ*_ (x = 0, 0.1 and 0.2). The asterisks correspond to a tiny impurity of Sr8(Al_12_O_24_)(MoO_4_)_2_ with sodalite structure (space group *I* 4_1_/*acd*).

A first identification of the SrMo_0.9_Al_0.1_O_3−*δ*_ and SrMo_0.8_Al_0.2_O_3−*δ*_ phases and structures was carried out by XRD at room temperature (Figure 1). SrMo_0.9_Al_0.1_O_3−*δ*_ was obtained as a pure perovskite whereas a tiny impurity of Sr_8_(Al_12_O_24_)(MoO_4_)_2_ (with Sodalite structure) was found in the sample with 20% of Al. In order to have a reference, undoped SrMoO_3_ was also prepared under the same synthesis conditions, yielding a cubic perovskite with *Pm*-3*m* space group and unit-cell parameter of *a* = 3.9760(3) Å. SrMo_0.9_Al_0.1_O_3−*δ*_ and SrMo_0.8_Al_0.2_O_3−*δ*_ phases exhibit the same space group as the undoped sample. The unit-cell parameters were determined by the Rietveld method as *a* = 3.9589 (4) and 3.9521 (8) Å for x = 0.1, 0.2, respectively. There is a conspicuous contraction of the lattice as the amount of Al increases, due to the smaller ionic size of Al^3+^ (0.535 Å) compared to the Mo^4+^ (0.65 Å) in octahedral coordination [22].

**Figure 2:**
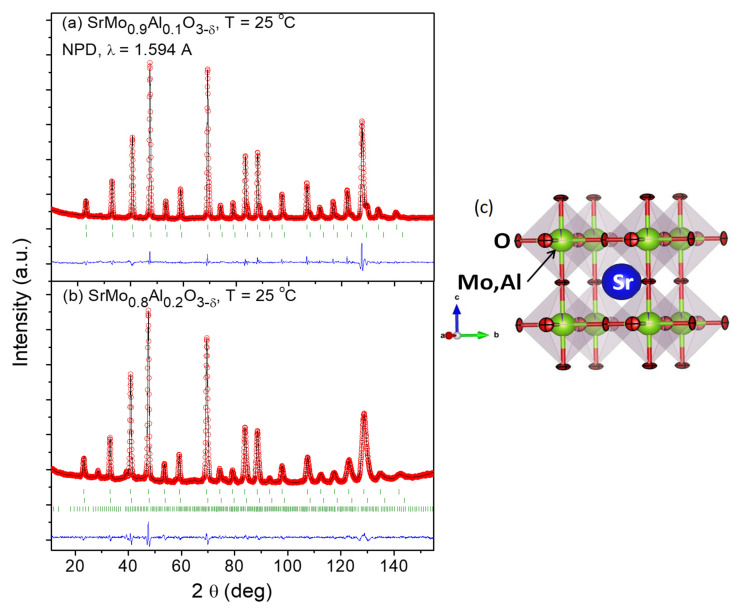
Observed (crosses), calculated (full line), and difference (at the bottom) NPD profiles for (**a**) SrMo_0.9_Al_0.1_O_3−_*_δ_* and (**b**) SrMo_0.8_Al_0.2_O_3−_*_δ_* at 25 °C, refined in the cubic *Pm*-*3m* space group. The vertical markers indicate the allowed Bragg reflections. The second series of Bragg reflections corresponds to a second perovskite phase with a higher amount of Al. The third series for x = 0.2 belong to Sr_8_(Al_12_O_24_)(MoO_4_)_2_ with sodalite structure. (**c**) View of the cubic perovskite crystal structure, where the blue sphere is the Sr atom, the red spheres are the oxygens and the green spheres are the Mo, Al atoms.

Due to the weak scattering factor for O^2−^ ions in perovskite oxides, an NPD study at RT was necessary in order to determine O positions and occupancy, in both scheelite (oxidized) and perovskite (reduced) phases. Figure 2a,b show the good agreement between the observed and calculated NPD patterns at room temperature for the SrMo_1−x_Al_x_O_3−*δ*_ (x = 0.1, 0.2) crystal structures, confirming the cubic symmetry with unit-cell parameters *a* = 3.95893 (8) and 3.9435 (2) Å, respectively.

Figure 2c shows the cubic perovskite structure that was refined by Rietveld method in the *Pm*-3*m* space group (No. 221), Z = 1, with Sr atoms located at 1*b* (½, ½, ½) sites, Mo and Al atoms distributed at random at 1*a* (0, 0, 0) sites, and the O oxygen atoms located at 3*d* (½, 0, 0) positions. During the refinement, the presence of shoulders in the high-angle reflections suggested the occurrence of second perovskite phases with slightly lower unit-cell parameters, due to the incorporation of higher amounts of Al. This phase segregation, due to an inhomogeneous distribution of the doping element, has also been observed in other systems, such as the skutterudites La_x_Co_4_Sb_12_ [23] or Ce_x_CoSb_12_ [24]. In our case, the second perovskite phases exhibited correspondingly smaller lattice parameters (3.9317 (2) Å and 3.9208 (5) Å for x = 0.1 and 0.2, respectively). These second phases were not observed by XRD (Figure 1), and they correspond to the second series of Bragg reflections displayed in Figure 2a,b. From the NPD scale factors, SrMo_1−x_Al_x_O_3−*δ*_ present 82% and 83% of the main phase, for x = 0.1, 0.2, respectively.

The Mo/Al and O occupancies were refined; interestingly, the main phases show an Al content lower than the nominal one, and in both cases, a full oxygen stoichiometry is observed. For instance, the crystallographic formulae are SrMo_0.975(1)_Al_0.025(1)_O_2.991(1)_ and SrMo_0.873(1)_Al_0.127(1)_O_3.003(2)_, for the x = 0.1 and x = 0.2 materials at room temperature. This is compatible with the Al-rich phase observed in the x = 0.2 pattern, Sr_8_(Al_12_O_24_)(MoO_4_)_2_ with sodalite structure. Moreover, the minority phase contains a significantly larger amount of Al; for instance, for x = 0.2 the Al contents is 0.48(1). The anisotropic displacement factors of O were also refined (constrained in both phases) and are included in Table 1. They are also shown in Figure 2c, presenting flattened ellipsoids that correspond to the highly covalent Mo-O bonds.

As observed by XRD, the unit-cell parameters and <Mo/Al-O> bond lengths decrease at room temperature with Al-doping, due to the smaller ionic size of Al^3+^ (0.535 Å) compared to the Mo^4+^ (0.65 Å) [22].

It is remarkable that the oxygen stoichiometry of the major perovskite phases is close to the expected value of 3.00, despite the introduction of a lower-valence element, such as Al, into the Mo^4+^ positions. The reason behind this finding is probably due to the valence variability of Mo being able to adopt a mixed Mo^4+^-Mo^5+^ valence to accommodate the introduction of Al (hole doping effect). For instance, a mixed valence of Mo^4.14+^, implying 14%Mo^5+^ + 86%Mo^4+^ would correspond to the mentioned crystallographic stoichiometry SrMo_0.873(1)_Al_0.127(1)_O_3.00_ for the nominal x = 0.2 compound, at room temperature.

**Figure 3:**
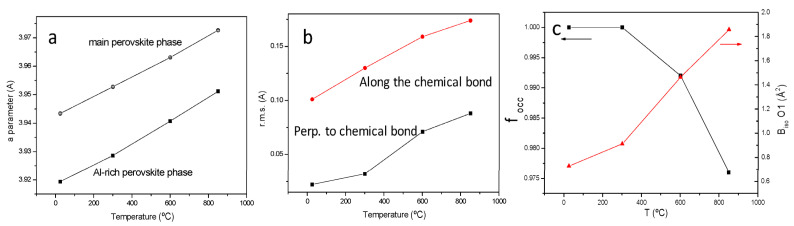
(**a**) Thermal evolution of the *a* unit-cell parameter of the main and minor perovskite phases, showing a similar expansion rate, (**b**) r.m.s. of the disk-shaped anisotropic displacement parameters, with the short axis along the Mo-O chemical bond, and the large axis (disk diameter) perpendicular to the chemical bond. (**c**) Evolution of the oxygen contents (left axis) of the main perovskite phase and B_eq_ displacement factor (right axis).

It is interesting to evaluate the evolution of the oxygen contents with temperature in order to assess the presence of oxygen vacancies at the working temperature of these anode materials within a SOFC. For this purpose, a temperature dependent NPD study was performed for the x = 0.2 compound, collecting additional NPD patterns at 300, 600, and 850 °C within a vanadium furnace working under vacuum coupled to the neutron diffractometer. The crystal structure remains cubic in the entire temperature interval, and it could be refined with the same crystallographic model, in the *Pm-3m* space group. Figure 3a displays the thermal evolution of the unit-cell parameter for the main and minor (Al-rich phase) perovskites, which regularly increase with temperature, with a similar rate, as expected. A thermal expansion coefficient (TEC) of 9.01 × 10^−6^ K^−1^ is estimated between 25 and 850 °C for the main perovskite phase. The disk-shaped anisotropic displacements are characterized by the magnitude of the r.m.s. (root mean square) parameters of the ellipsoid semiaxes. Figure 3b shows the r.m.s for the short semiaxis (along the Mo-O chemical bond) and the long semiaxis (perpendicular to it); the flattened ellipsoids become progressively less anisotropic as temperature increases, which might be related to the oxygen diffusion enhancement as the oxygen vacancies are generated. Indeed, the occupancy factor of oxygen positions diminishes with temperature as shown in Figure 3c (left axis). At 850 °C, 2.3% of oxygen vacancies are determined, assessing the oxygen defective nature of this perovskite at the working conditions of a SOFC. Figure 3c (right axis) also shows the equivalent anisotropic displacement factors of oxygen atoms (B_eq_) increasing from 0.727 Å^2^ at 25 °C to 1.858 Å^2^ at 850 °C, which suggest the oxygen disorder necessary for the pertinent behaviour of a MIEC electrode.

**Figure 4:**
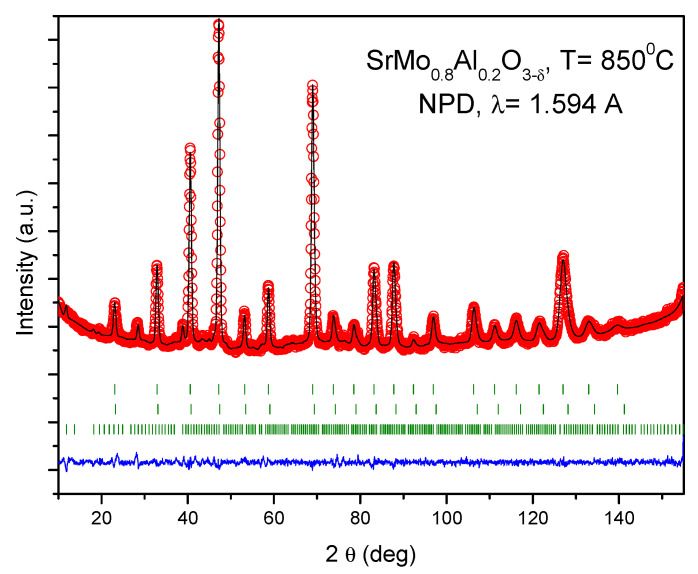
Rietveld plot for the reduced SrMo_0.8_Al_0.2_O_3−_*_δ_* perovskite phase. Observed (crosses), calculated (full line) and difference (at the bottom) NPD profiles at 850 °C, refined in the cubic *Pm-3m* space group. The vertical markers indicate the allowed Bragg reflections. The second series of Bragg reflections correspond to a second perovskite phase with a higher amount of Al. The third series belongs to Sr_8_(Al_12_O_24_)(MoO_4_)_2_ with sodalite structure.

A Rietveld plot of the NPD pattern at the maximum temperature of 850 °C is displayed in Figure 4. Table 2 includes the structural parameters after the refinement of the SrMo_0.8_Mg_0.2_O_3−*δ*_ structure at the different temperatures under study.

### 3.2. Thermal Expansion Measurements

**Figure 5:**
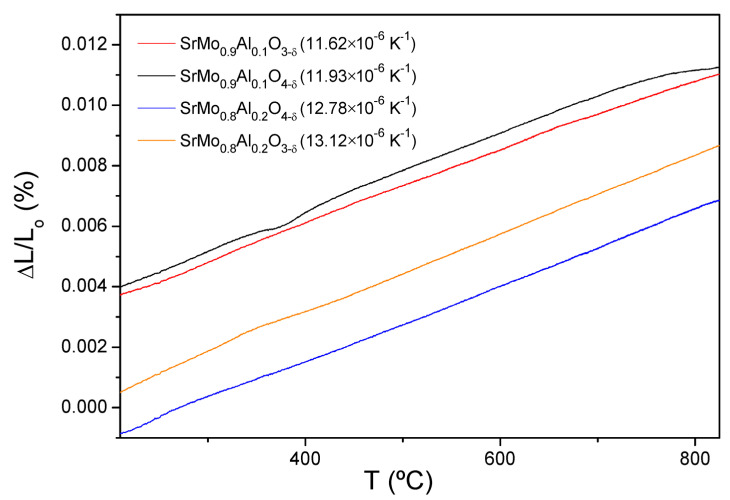
Thermal expansion determined by dilatometry of the SrMo_1−x_Al_x_O_3−*δ*_ and SrMo_1__−__x_Al_x_O_4__−*δ*_ series.

The temperature dependencies of the thermal expansion rates of SrMo_1−x_Al_x_O_3−*δ*_ (x = 0.1 and 0.2) perovskites and SrMo_1−x_Al_x_O_4−_*_δ_* (x = 0.1 and 0.2) scheelites, measured in 5% H_2_ and air atmospheres, respectively, are shown in Figure 5. The analysis was carried out for several cycles and the temperature range chosen was from 200 to 850 °C, only recording the data during heating. The thermal expansion curves in Figure 5 display a monotonic behaviour in the measured temperature range, with no abrupt changes for any composition. This analysis was carried out to demonstrate that these anode materials present similar TEC values to the buffer layer and the electrolyte; this is an essential requirement for the good performance of the cell, avoiding cracking problems upon thermal cycling.

In this case, both reduced and oxidized materials have very similar TEC values as shown in Figure 5 (between 11.62 and 13.12 × 10^−6^ K^−1^). These coefficients are also comparable to the LSGM (12.50 × 10^−6^ K^−1^ [26]) and LDC (13.4 × 10^−6^ K^−1^ [27]) TEC values, and thus, a stress at the interface of the cell during the oxidation-reduction cycles is avoided.

### 3.3. Chemical Compatibility

**Figure 6:**
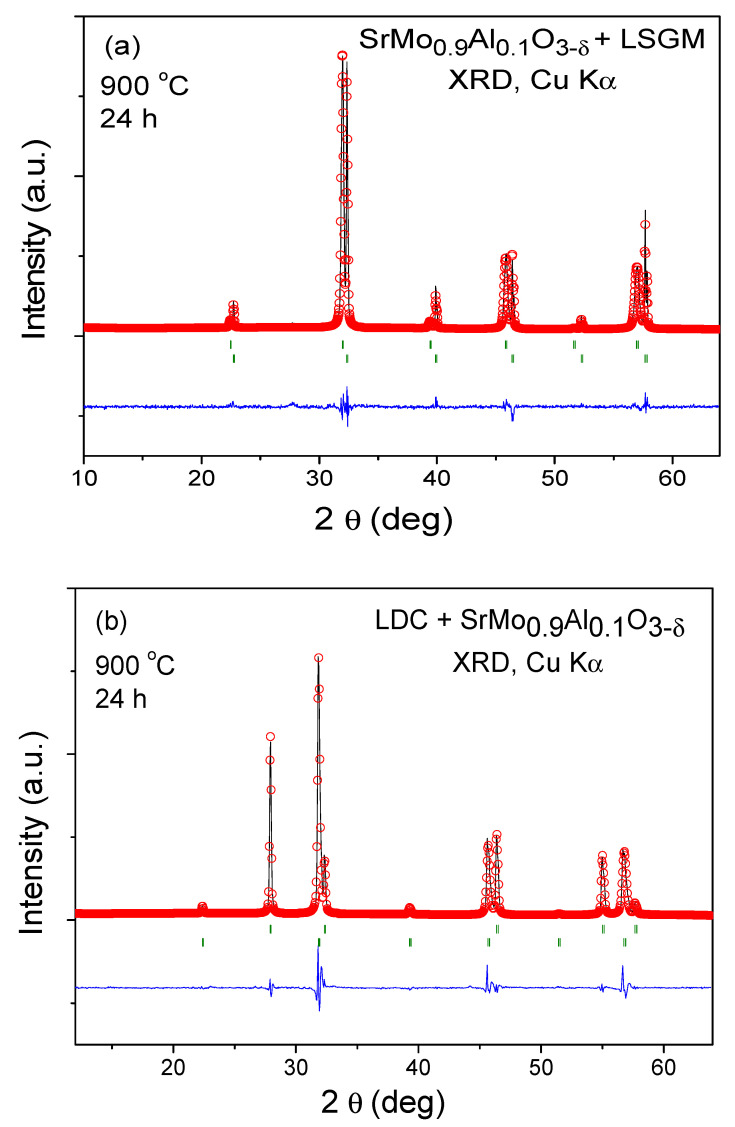
Rietveld-refined XRD profiles of a mixture of (**a**) SrMo_0.9_Al_0.1_O_3__−*δ*_ and LSGM and (**b**) LDC and SrMo_0.9_Al_0.1_O_3__−*δ*_ after a thermal treatment at 900 °C in H_2_(5%)/N_2_, showing no reaction products between both phases other than the initial reactants. The first and second series of Bragg positions correspond to (**a**) SrMo_0.9_Al_0.1_O_3__−*δ*_ and LSGM and (**b**) LDC (with fluorite structure) and SrMo_0.9_Al_0.1_O_3__−*δ*_, respectively.

The chemical compatibility of SrMo_1−x_Al_x_O_3−*δ*_ (x = 0.1 and 0.2) with the LSGM electrolyte and LDC buffer layer was studied. Equal quantities of SrMo_1−x_Al_x_O_3−*δ*_ (x = 0.1)/LSGM and SrMo_1−x_Al_x_O_3−*δ*_ (x = 0.1)/LDC were mixed and calcined at 900 °C for 24 h in 5%H_2_ atmosphere to avoid the oxidation of the perovskite. After these treatments, a XRD analysis was carried out to check if secondary phases appeared in the diffractogram. Figure 6a,b show the resulting XRD patterns analyzed using the Rietveld method: no extra peaks are observed in any cases, excluding both starting phases. This study demonstrates that SrMo_1−x_Al_x_O_3−*δ*_ (x = 0.1)/LSGM and SrMo_1−x_Al_x_O_3−*δ*_ (x = 0.1)/LDC are chemically compatible. The same procedure was applied to the SrMo_1−x_Al_x_O_3−*δ*_ (x = 0.2) perovskite obtaining the same result.

### 3.4. Thermal Analysis

**Figure 7:**
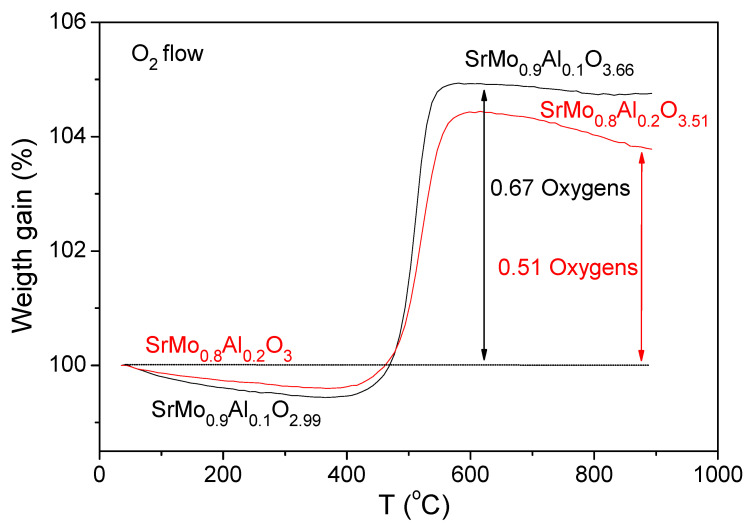
Thermal analysis curves for the SrMo_0.9_Al_0.1_O_2.99_ and SrMo_0.8_Al_0.2_O_3_ samples in O_2_ flux.

The oxygen content variation of SrMo_0.9_Al_0.1_O_3__−_*_δ_* and SrMo_0.8_Al_0.2_O_3__−_*_δ_* samples with temperature was evaluated by a thermogravimetric analysis in O_2_ flow from 35 to 900 °C. Figure 7 shows the TG curves, where the reduced SrMo_0.9_Al_0.1_O_2.99_ and SrMo_0.8_Al_0.2_O_3_ perovskites are oxidized between 450 and 550 °C, incorporating 0.67 and 0.51 oxygen atoms per formula, respectively. After the oxidation of both samples, two deficient scheelite materials with SrMo_0.9_Al_0.1_O_3.66_ and SrMo_0.8_Al_0.2_O_3.51_ compositions were obtained. The Mo final valence was 5.6+ for x = 0.1 and 5.5+ for x = 0.2.

**Figure 8:**
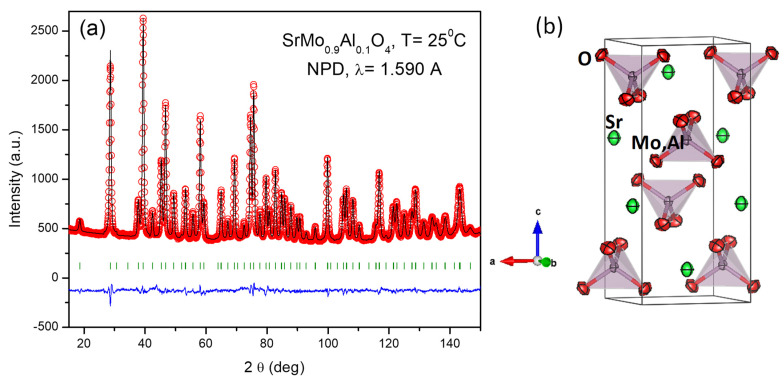
(**a**) Rietveld plot after the structural refinement from NPD data of the oxidation product yielding SrMo_0.9_Al_0.1_O_3.66_ scheelite. Observed (crosses), calculated (full line), and difference (at the bottom) NPD profiles in the tetragonal I4/*a* space group. The vertical markers indicate the allowed Bragg reflections. (**b**) View of the scheelite crystal structure, after the full anisotropic refinement of the displacement factors.

After the thermogravimetric analysis, an NPD study of SrMo_0.9_Al_0.1_O_3.66_ was carried out to check if the oxidation of the sample was completed. Figure 8 displays a Rietveld plot after the structural refinement of the sample in which a pure scheelite was obtained. This scheelite structure was refined in the *I*4_1_*/a* (No. 88) space group, where Sr atoms are situated at the 4*b* (0, ¼, ⅝) positions; Mo and Al atoms are randomly distributed at 4*a* (0, ¼, ⅛) sites; and O oxygen atoms are located at the 16*f* (x, y, z) positions. A full anisotropic refinement of the displacement factors was carried out for all the atoms. Figure 8 exhibits the Rietveld plot after the refinement, showing an excellent agreement between observed and calculated profiles. Figure 8 also includes a view of the structural arrangement, which can be described as a superstructure of fluorite formed by two different groups, one of them with Mo ions coordinated by four O^2−^ anions in tetrahedral coordination [MoO_4_], and the other one formed by Sr cations in eightfold oxygen coordination [SrO_8_]. Table 3 contains the crystallographic parameters after the refinement from NPD data; there is a conspicuous oxygen deficiency corresponding to the Al doping at the Mo sites.

Finally, the scheelite sample was introduced in a tubular furnace and was heated at 1050 °C for 15 h in a 5% H_2_ atmosphere. After this thermal treatment, the initial perovskite structure is recovered, thus confirming reversibility upon cycling in oxidizing/reducing atmospheres, which is an essential requirement in these materials.

### 3.5. Electrical Conductivity Measurements

**Figure 9:**
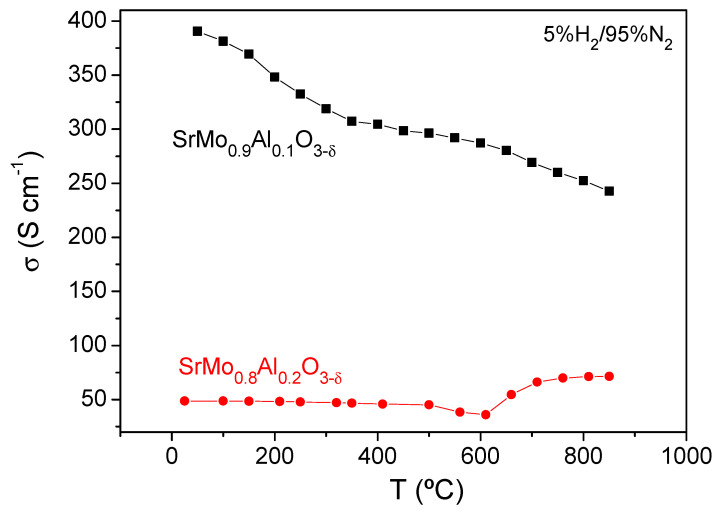
The dc-conductivity as a function of temperature for SrMo_1__−_Al_x_O_3__−*δ*_ (x = 0.1 and 0.2).

The thermal variation of the electrical conductivity of SrMo_1−x_Al_x_O_3−*δ*_ (x = 0.1 and 0.2) is shown in Figure 9. Under reducing conditions, a metallic-like conductivity is observed in case of SrMo_0.9_Al_0.1_O_3__−*δ*_ in all the range of temperatures measured, with a maximum value of σ = 243 Scm^−1^ at 850 °C. However, SrMo_0.8_Al_0.2_O_3−*δ*_ sample shows a metallic behavior from 20 to 600 °C, and a metal-to-insulator transition is observed at this temperature, with a positive slope suggesting a semiconducting behaviour above 600 °C. The maximum conductivity obtained at 850 °C is 70 Scm^−1^ for SrMo_0.8_Al_0.2_O_3−*δ*_. It is observed that the electrical conductivity decreases when the Al content increases, since Al^3+^ perturbs the conduction paths via Mo-O-Mo chemical bonds. This can also be the origin of the change in the slope of SrMo_0.8_Al_0.2_O_3−*δ*_ at 600 °C, which triggers an electron hopping mechanism when the crystal lattice is expanded with temperature. Even so, the electrical conductivity values obtained at 850 °C for both materials are sufficiently high for the correct performance of these oxides as anodes in SOFC, and is comparable with those described for other anode materials such as SrMo_0.9_Mg_0.1_O_3−*δ*_ [16] and SrMo_0.9_Ga_0.1_O_3−*δ*_ [17] that have been successfully used in SOFCs with an extraordinary performance.

### 3.6. Fuel-Cell Evaluation

**Figure 10:**
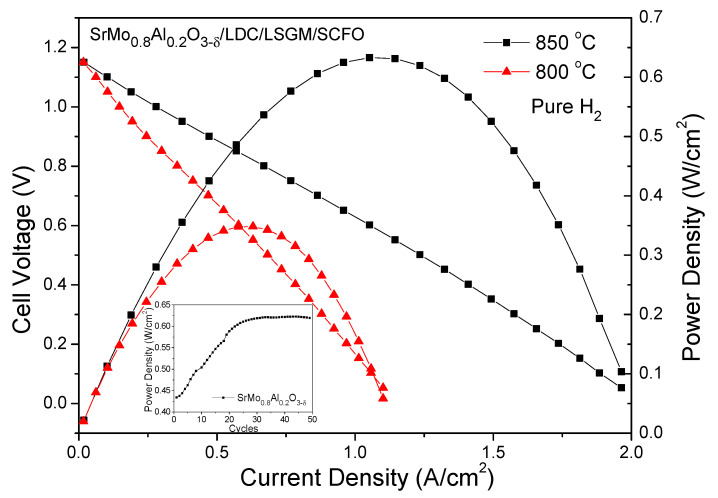
Cell voltage (left axis) and power density (right axis) as a function of the current density for the test cell with the configuration of SrMo_0.8_Al_0.2_O_3−*δ*_/LDC/LSGM/SCFO measured in pure H_2_ at T = 800 and 850 °C. The inset in Figure 7 shows the evolution of the power density as a function of the number of cycles at 850 °C for SrMo_0.8_Al_0.2_O_3−*δ*_ single cell.

To check the performance of SrMo_1−x_Al_x_O_3__−*δ*_ anode materials, a single cell in an electrolyte-supported set up using a 300 µm-thick LSGM electrolyte with SrMo_1−x_Al_x_O_3−*δ*_(x = 0.2)/LDC/LSGM/SrCo_0.8_Fe_0.2_O_3−*δ*_ configuration was prepared. A hydrogen flow was used to feed the anode of the SOFC, whereas the cathode was opened to the air. Figure 10 shows high OCV of 1.15 V, meaning a good sealing of the cell and a dense electrolyte. Once the current starts to increase, the internal resistance of the cell accounts for the observed voltage decrease. Finally, the product of the cell voltage times the current density equals the power density, giving a maximum value of 633 mW/cm^2^ for x = 0.2 (right axis of Figure 10).

The inset in Figure 10 corresponds to the maximum power density as a function of the number of cycles for SrMo_0.8_Al_0.2_O_3−*δ*_. As the anode material is being reduced from the scheelite to the perovskite phase, the power density increases. This process occurs during the first 25 cycles, after which the power density reaches a plateau, and it remains stable at 633 mW/cm^2^.

As shown in Table 4, this result exceeds, in most cases, those obtained for other similar systems such as SrMo_0.8_M_0.2_O_3−*δ*_ (M = Cr, Co, Fe, Mg, Ga) at 850 °C and confirms the excellent performance of this material as an anode in SOFCs.

### 3.7. Scanning Electron Microscopy

**Figure 11:**
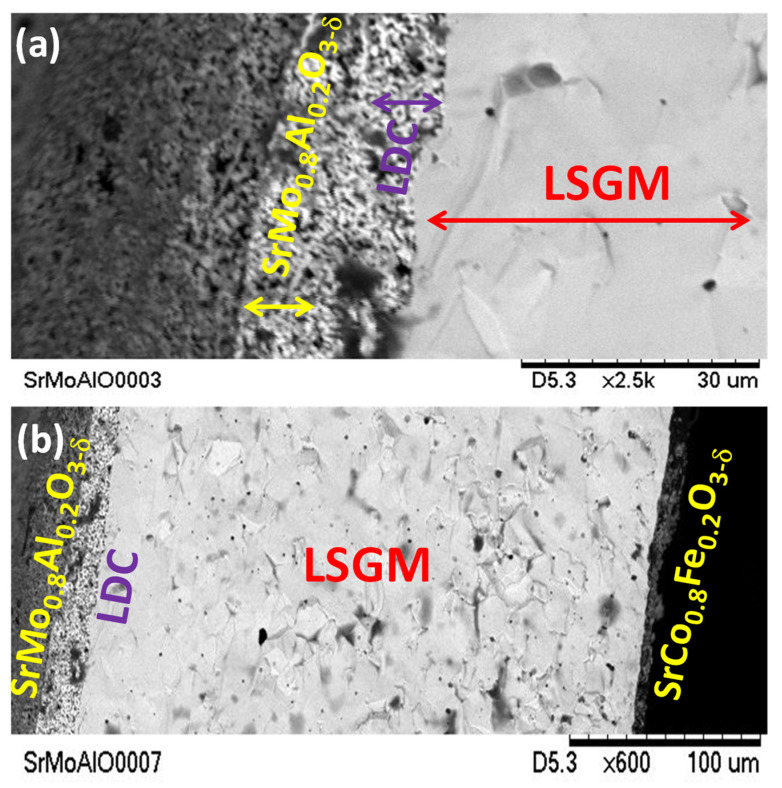
SEM micrographs of (**a**) SrMo_0.8_Al_0.2_O_3−*δ*_ sample showing the porous anode and buffer layers deposited on one side of dense LSGM electrolyte and (**b**) interface SMAO/LDC/LSGM/SCFO after the single cell test.

Figure 11a,b present two SEM micrographs of a post-mortem single cell formed by SrMo_0.8_Al_0.2_O_3−*δ*_/LDC/LSGM/SrCo_0.8_Fe_0.2_O_3−*δ*_ materials, showing the different layers of the cell. A dense LSGM electrolyte pellet is observed, with a thickness of 300 µm. Both porous layers of LDC and SrMo_0.8_Al_0.2_O_3−*δ*_ present around a 10 µm width and were deposited onto one side of the LSGM. A thinner and porous SrCo_0.8_Fe_0.2_O_3−*δ*_ layer of 5 µm was deposited on the other side of the LSGM pellet (Figure 11b). It is also shown, in this post-mortem test, that the electrodes are homogenously adhered to the electrolyte, with no symptoms of delamination.

**Figure 12:**
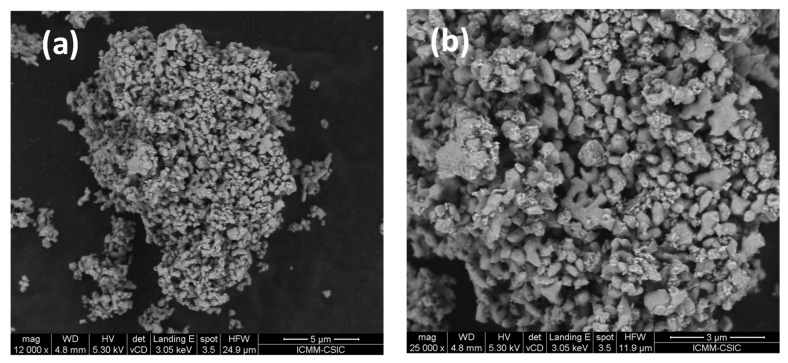
(**a**) 12,000× and (**b**) 25,000× SEM micrographs of SrMo_0.9_Al_0.1_O_3−*δ*_ sample showing an excellent porosity. The typical particle size is below 1µm, although groups of several particles sintered together are identified.

The porosity of the SrMo_0.9_Al_0.1_O_3−*δ*_ can also be assessed in Figure 12a,b where the morphology of the anode in powder form is shown. These two pictures illustrate an excellent porosity with a pore size of around 1 µm. This excellent porosity is an essential condition that electrode materials must exhibit, in order to favour the gas flow across the grains.

Figure S1 in the Supplementary Information (SI) shows the EDX spectrum where the major occurrence of Sr, Mo, Al, and O is observed. On the other hand, the table in Figure S1 indicates the % weight of Sr, Mo, Al, and O in the material, in good agreement with those expected.

## 4. Conclusions

SrMo_1−x_Al_x_O_3−*δ*_ perovskite oxides are proposed as anode materials for SOFCs. The synthesis by soft chemistry procedures leads to oxidized scheelite phases (SrMo_1−x_Al_x_O_4−*δ*_), which are reduced in H_2_ flow to give the active perovskite oxides. An NPD study demonstrates that, although the perovskite phases are virtually oxygen stoichiometric at RT conditions, they become oxygen deficient as temperature is increased to the working conditions of a SOFC, thus incorporating the required defects for its MIEC performance. A single test cell set as SrMo_1−x_Al_x_O_3−_*_δ_*/LDC/LSGM/SrCo_0.8_Fe_0.2_O_3−_*_δ_* (x = 0.2) in an electrolyte-supported conformation was measured using pure H_2_ as a fuel, yielding an output power of 633 mW/cm^2^ at 850 °C. The TEC value is demonstrated to be compatible to the other components of the cell. A chemical compatibility with the LSGM electrolyte and LDC buffer layer and a good reversibility between the oxidized and reduced phases, as well as the porosity and adherence of the deposited electrodes are also verified. All these features are well accomplished and, hence, these reduced perovskite oxides may be considered as potential anodes for SOFCs.

## Figures and Tables

**Table 1:** Unit-cell and thermal parameters for SrMo_1−x_Al_x_O_3−_*_δ_* (x = 0, 0.1 and 0.2) in cubic *Pm-3m* (no. 221) space group, from NPD at RT, corresponding to the major perovskite phase. Sr is placed at 1*b* (1/2, 1/2, 1/2), (Mo,Al) at 1*a* (0, 0, 0), and O at 3*d* (1/2, 0, 0) position.

*SrMo_1−x_Al_x_O_3−δ_*	*x = 0 ^a^*	*x = 0.1*	*x = 0.2*
***a (Å)***	*3.97629(3)*	*3.95893(8)*	*3.9438(2)*
***V(Å)^3^***	*62.869(7)*	*62.049(2)*	*61.342(4)*
***Sr 1b (½, ½, ½)***			
***B_iso_ (Å^2^)***	*0.77(3)*	*0.743(3)*	*0.95(1)*
***f_occ_***	*1.00*	*1.00*	*1.00*
***Mo/Al 1a (0, 0, 0)***			
***B_iso_ (Å^2^)***	*0.55(4)*	*0.198(3)*	*0.35(1)*
***Mo/Al f_occ_***	*1.00*	*0.97 5(1)/0.025(1)*	*0.874/0.127*
***O1 3d (½, 0, 0)***			
β_11_ *	*-*	*30(7)*	*34(8)*
β_22_ *	*-*	*159(5)*	*159(6)*
β_33_ *	*-*	*159(5)*	*159(6)*
***B_eq_ (Å^2^)***	*0.75(10)*	*0.7273*	*0.6560*
***f_occ_***	*1.00*	*0.997(1)*	*1.001(1)*
***Reliability factors***			
***χ^2^***	*-*	*5.84*	*1.52*
***R_p_(%)***	*-*	*3.71*	*1.71*
***R_wp_ (%)***	*-*	*5.02*	*2.18*
***R_exp_(%)***	*-*	*2.07*	*1.77*
***R_Bragg_(%)***	*-*	*2.85*	*1.80*
***Distances (Å)***			
***(Sr)-(O1)***	*-*	*2.79939(4)*	*2.7873(4)*
***(Mo/Al)-(O1)***	*1.98814(1)*	*1.97947(4)*	*1.9719(4)*

^a^ Taken from Ref. [25], * Anisotropic Betas (×10^4^). β_12_ = β_13_ = β_23_ = 0.

**Table 2:** Unit-cell, thermal parameters, and selected distances (Å) for SrMo_0.8_Al_0.2_O_3−_*_δ_* (major perovskite phase) refined in the cubic *Pm-3m* (no. 221) space group, from NPD from 300 to 850 °C.

*SrMo_0.8_Al_0.2_O_3−δ_*	*300 °C*	*600 °C*	*850 °C*
***a (Ǻ)***	*3.9527(2)*	*3.9631(3)*	*3.9722(4)*
***V(Ǻ)^3^***	*61.758(6)*	*62.245(8)*	*62.68(1)*
***Sr 1b (½, ½, ½)***			
***B_iso_ (Å^2^)***	*1.156(7)*	*1.739(8)*	*2.25(1)*
***f_occ_***	*1.00*	*1.00*	*1.00*
***Mo/Al 1a (0, 0, 0)***			
***B_iso_ (Å^2^)***	*0.307(6)*	*0.488(7)*	*0.659(9)*
***Mo/Al f_occ_***	*0.874/0.127*	*0.874/0.127*	*0.874/0.127*
***O1 3d (½, 0, 0)***			
β_11_ *	*13(13)*	*64(15)*	*153(20)*
β_22_ *	*212(10)*	*317(12)*	*365(15)*
β_33_ *	*212(10)*	*317(12)*	*365(15)*
***B_eq_ (Å^2^)***	*0.9111*	*1.4624*	*1.8578*
***f_occ_***	*1.00*	*0.992(4)*	*0.976*
***Reliability factors***			
***χ^2^***	*1.92*	*1.84*	*1.44*
***R_p_(%)***	*1.91*	*1.87*	*1.78*
***R_wp_ (%)***	*2.53*	*2.55*	*2.27*
***R_exp_(%)***	*1.83*	*1.88*	*1.89*
***R_Bragg_(%)***	*3.13*	*2.20*	*1.62*
***Distances (Å)***			
***(Sr)-(O1)***	*2.7950(1)*	*2.8023(2)*	*2.8088(2)*
***(Mo/Al)-(O1)***	*1.9764(1)*	*1.9816(2)*	*1.9861(2)*

* Anisotropic Betas (×10^4^). β_12_ = β_13_ = β_23_ = 0

**Table 3:** Structural parameters of SrMo_0.9_Al_0.1_O_3.67_ scheelite after the Rietveld refinement from NPD data.

*Crystal Data*						
*Tetragonal, I4_1_/a*	*NPD, λ = 1.594 Å*					
*a = 5.37903 (9) Å*	*c = 12.0190(3) Å*					
*V = 347.76 (1) Å3*	*Z = 4*					
***Refinement***						
R_p_ = 1.98%	R_wp_ = 2.56%					
R_exp_ = 1.35%	R_Bragg_ = 3.85%					
χ^2^ = 3.76	3199 data points					
Fractional atomic coordinates and equivalent isotropic displacement parameters (Å^2^)						
	***x***	***y***	***z***	***U_eq_***	**Occ. (<1)**	
***Sr***	*0*	*0.25*	*0.625*	*0.0127(9)*		
***Mo***	*0*	*0.25*	*0.125*	*0.0054(9)*	*0.9*	
***Al***	*0*	*0.25*	*0.125*	*0.0054(9)*	*0.1*	
***O***	*0.2370(2)*	*0.11321(16)*	*0.04330(10)*	*0.0135(5)*	*0.9841(7)*	
Atomic displacement parameters (Å^2^)						
	*U* ^11^	*U* ^22^	*U* ^33^	*U* ^12^	*U* ^13^	*U* ^23^
***Sr***	0.0118(6)	0.0118(6)	0.0143(14)	0	0	0
***Mo***	0.0050(6)	0.0050(6)	0.0061(13)	0	0	0
***Al***	0.0050(6)	0.0050(6)	0.0061(13)	0	0	0
***O***	0.0144(5)	0.0134(4)	0.0127(4)	0.0044(5)	0.0038(3)	−0.0011(5)

**Table 4:** Power density values for other similar systems such as SrMo_0.8_M_0.2_O_3−*δ*_ (M = Cr, Co, Fe, Mg, Ga) anodes at 850 °C.

Anode Material	Power Density
**SrMo_0.8_Cr_0.2_O_3−*δ*_**	527 mW cm^−2^ [15]
**SrMo_0.8_Co_0.2_O_3−*δ*_**	545 mW cm^−2^ [18]
**SrMo_0.8_Fe_0.2_O_3−*δ*_**	551 mW cm^−2^ [14]
**SrMo_0.8_Mg_0.2_O_3−*δ*_**	684 mW cm^−2^ [16]
**SrMo_0.8_Ga_0.2_O_3−*δ*_**	560 mW cm^−2^ [17]
**SrMo_0.8_Al_0.2_O_3−*δ*_**	633 mW cm^−2^ [material of this work]

## Supplementary Materials

The following supporting information can be downloaded at: https://www.mdpi.com/article/10.3390/ma15113819/s1, Figure S1: Upper panel: SEM image where the EDX spectrum was collected, and relative contents of Sr, Mo, Al and O. Lower panel: typical EDX spectrum.
