# Supplementary material for: Al-Doped SrMoO3 Perovskites as Promising Anode Materials in Solid Oxide Fuel Cells
Source: Materials (Basel). 2022 May 27;15(11):3819. doi: 10.3390/ma15113819 (PMC9181521; doi:10.3390/ma15113819)
Supplement: Supplementary file 1 [file materials-15-03819-s001.zip › materials-1725516-supplementary.pdf]

# Supporting Information

# Al-doped $SrMoO_3$ Perovskites as promising anode materials in Solid Oxide Fuel Cells

V. Cascos[a,b]*, M.T. Fernández-Díaz,[c] J.A. Alonso[a],

[a] *Instituto de Ciencia de Materiales de Madrid, C.S.I.C., Cantoblanco E-28049 Madrid*
[b]*Departamento de Química Inorgánica, Universidad Complutense de Madrid, E-28040, Madrid, Spain*
[c] *Institut Laue Langevin, BP 156X, Grenoble, F-38042, France*

*vcascos@ucm.es

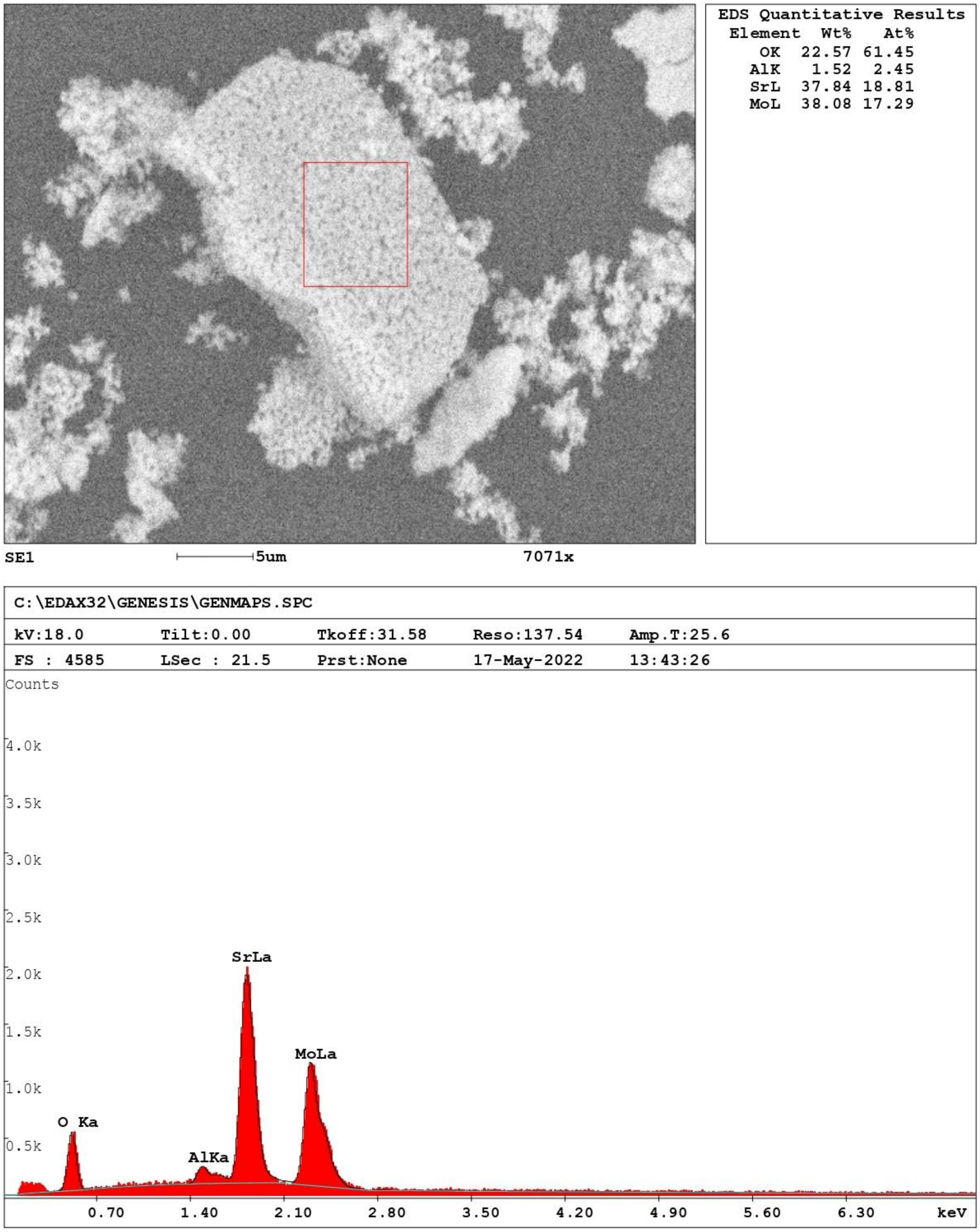


**Figure S1.** Upper panel: SEM image where the EDX spectrum was collected, and relative contents of Sr, Mo, Al and O. Lower panel: typical EDX spectrum.
